# 236. Insight of Polymicrobial Prosthetic Joint Infections at a Referral Hospital

**DOI:** 10.1093/ofid/ofab466.438

**Published:** 2021-12-04

**Authors:** Diana Fernández-Rodríguez, María de Lourdes García-Hernández, Guillermo Cerón-González, Claudia Adriana Colín-Castro, Melissa Hernández-Durán, Mercedes Isabel Cervantes-Hernández, Luis Esaú López-Jácome, Noé Becerra-Lobato, Jaime Arturo Mondragón-Eguiluz, Edgar Samuel Vanegas-Rodríguez, Rafael Franco-Cendejas

**Affiliations:** 1 MD/PhD Plan de Estudios Combinados en Medicina (PECEM), Mexico City, Distrito Federal, Mexico; 2 Instituto Nacional de Rehabilitación “Luis Guillermo Ibarra Ibarra”, Mexico City, Distrito Federal, Mexico

## Abstract

**Background:**

Approximately one-third of the prosthetic joint infections (PJIs) are polymicrobial. They are difficult to treat and there is an urgent need of clinical evidence that help to guide current protocols. We aimed to define the clinical characteristics and outcomes of patients with polymicrobial PJI.

**Methods:**

We conducted a retrospective cohort study of patients with polymicrobial PJI treated at a referral hospital in Mexico City. Clinical data was retrieved and analyzed. Time to treatment failure, was evaluated for all cases.

**Results:**

We identified 166 patients with a polymicrobial PJI from July 2011 to October 2020. The median follow-up period was 3.24 years (IQR, 1.45-6.42). Fistulae (77.7%) and pain (76.5%) were frequent. Patients required a median of 2 (IQR, 1-3) hospitalizations and 3 (IQR, 1-5) surgeries. Relapse, reinfection, and amputation ocurred in 21.1% (35), 10.2% (17), and 7.2% (12) of the cases, respectively. At 1-year follow-up 38.47% (63) patients failed to control the infection. At 2 and 5-year follow-up this rate increased to 50% (83) and 68% (112), respectively. The main infecting microorganisms were *Staphylococcus epidermidis* (51.8%), *Enterococcus faecalis* (47.6%), and *Staphyloccocus aureus* (34.9%). Anaerobes were identified in 38 (22.9%) cases. At 1 and 5-year follow-up, 39.31% (34) and 71.1% (61) of patients with *S. epidermidis* experienced treatment failure. On the other hand, those with *S. aureus* showed lower rates (log-rank p-value=0.03): 24.85% (14) and 50% (29), accordingly. Patients affected by anaerobes and *E. faecalis* exhibited similar trends, between them (log-rank p-value=0.73).

Table1. Clinical findings of patients with polymicrobial PJI.

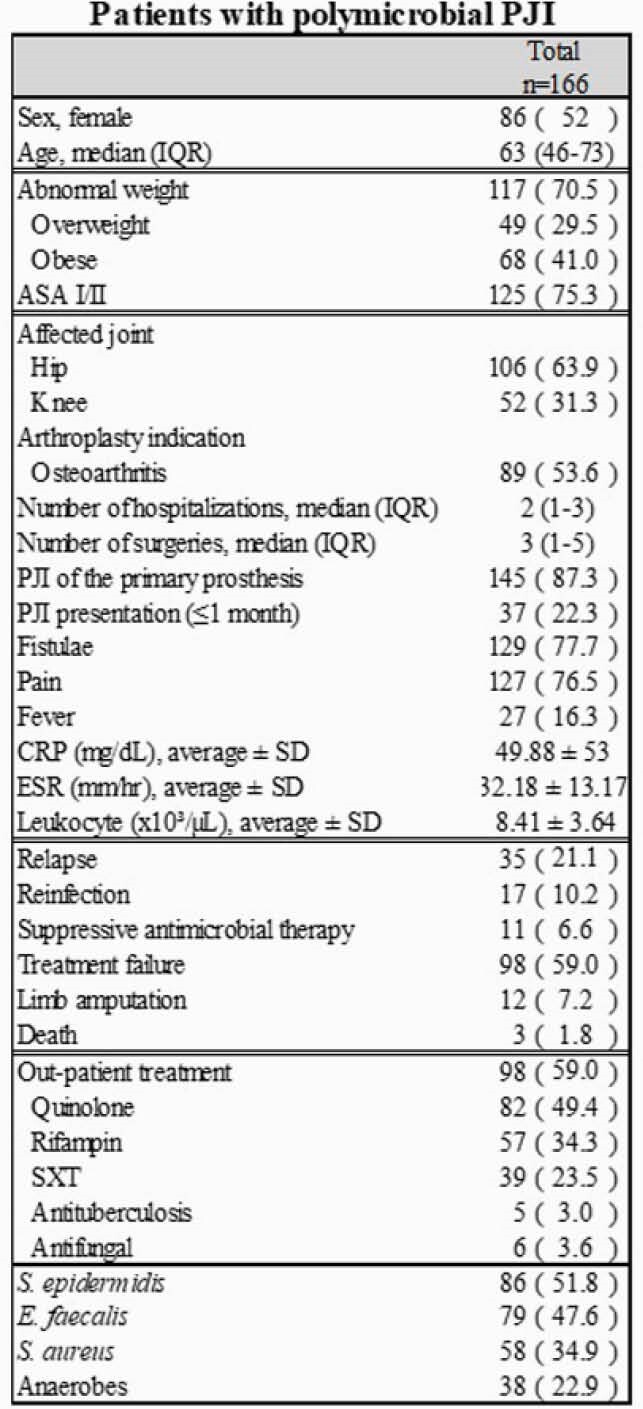

Frequency distributions of sociodemographic factors, comorbidities, clinical presentation, outcomes, out-patient treatment, and etiology in patients with polymicrobial PJI. Data is presented as absolute frequency followed by relative frequency enclosed in parenthesis, otherwise specified. Abbreviations: SXT, Trimethoprim/Sulfamethoxazole.

Figure 1. Kaplan‒Meier survivorship curve illustrating the time to treatment failure among patients with polymicrobial PJI. The shaded areas surrounding the gross line represent the 95% CI.

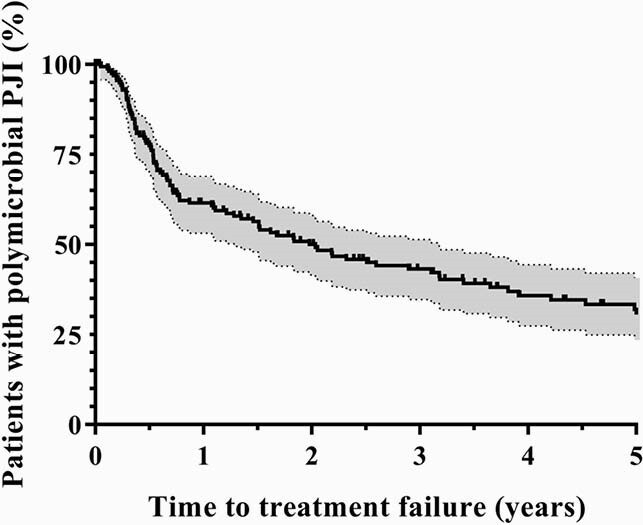

Figure2. Kaplan‒Meier survivorship curves illustrating the time to treatment failure among patients with polymicrobial PJI, according to the infecting microorganisms..

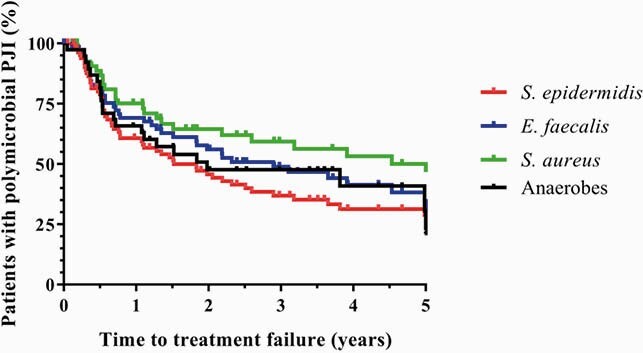

Patients affected by S. epidermidis, E.faecalis, S. aureus, and anaerobes are represented with red, blue, green, and black lines, respectively.

**Conclusion:**

Our study showed 61.53% of the patients with polymicrobial PJI controlled the infection at 1-year follow-up. This rate decreased over the years. These patients required a considerable number of hospitalizations and surgeries. Likewise, presenting with fistulae and pain ensured a high suspicion of PJI. *S. epidermidis, E. faecalis*, and *S. aureus* were the most frequent infecting microorganisms. The stratification of our cohort suggested the microbiology of polymicrobial PJI could have driven to differences in rates of treatment failure.

**Disclosures:**

**All Authors**: No reported disclosures

